# *Chlamydia* plasmid-encoded protein Pgp2 is a replication initiator with a unique β-hairpin necessary for iteron-binding and plasmid replication

**DOI:** 10.1128/iai.00602-24

**Published:** 2025-02-07

**Authors:** Danny Wan, Matthew Pan, Guangming Zhong, Huizhou Fan

**Affiliations:** 1 Department of Pharmacology, Rutgers Robert Wood Johnson Medical School 242612 , Piscataway, New Jersey, USA; 2 Department of Microbiology and Immunology, University of Texas Health San Antonio, San Antonio, Texas, USA; University of California Davis, Davis, California, USA

**Keywords:** *Chlamydia*, *Chlamydi*a plasmid, Pgp2, plasmid replication, replication initiator

## Abstract

The virulence plasmid of the obligate intracellular bacterium *Chlamydia* encodes eight proteins. Among these, Pgp3 is crucial for pathogenicity, and Pgp4 functions as a transcriptional regulator of both plasmid and chromosomal genes. The remaining proteins, Pgp1, Pgp5, Pgp6, Pgp7, and Pgp8, are predicted to play various roles in plasmid replication or maintenance based on their amino acid sequences. However, the function of Pgp2 remains unknown, even though it is required for transformation. In this study, we utilized AlphaFold to predict the three-dimensional (3-D) structure of *Chlamydia trachomatis* Pgp2. Despite a lack of apparent sequence homology, the AlphaFold structure exhibited high similarity to experimentally determined structures of several plasmid replication initiators. Notably, Pgp2 features a unique β-hairpin motif near the DNA-binding domain, absent in other plasmid replication initiators with overall 3-D structures similar to Pgp2. This β-hairpin motif is also present in AlphaFold models of Pgp2s across all 13 *Chlamydia* species. To assess its significance, we engineered a plasmid lacking the 11 amino acids constituting the β-hairpin motif in *C. trachomatis* Pgp2. Although this deletion did not alter the overall structure of Pgp2, the mutated plasmid failed to transform plasmid-free *C. trachomatis*. These findings reveal that Pgp2 is a plasmid replication initiator, with the β-hairpin motif playing a critical role in binding to its cognate iteron sequences in the replication origin of the chlamydial plasmid.

## INTRODUCTION

*Chlamydia* is an obligate intracellular bacterium pathogenic to humans and animals (1). For example, *Chlamydia trachomatis* is the leading cause of bacterial sexually transmitted infections globally and of preventable blindness in certain under-developed regions (2, 3). *Chlamydia muridarum* serves as a model for studying chlamydial diseases and immune responses in mice (4, 5).

All *Chlamydia* species carry a conserved plasmid. Although previously referred to as a “cryptic plasmid,” it is more accurately described as a virulence plasmid since studies involving mouse and non-human primate models demonstrated that plasmid-free *Chlamydia* strains exhibit significantly reduced pathogenicity (4–6). This highlights the possibility of targeting the plasmid to mitigate chlamydial infections.

The chlamydial plasmid encodes eight open reading frames, producing proteins designated Pgp1–8. Pgp3 is a key virulence factor, playing a crucial role in the progression of chlamydial infections from the lower genital tract to the upper genital tract (7–9) and in the colonization of the gastrointestinal tract (10). Pgp4 is a transcriptional regulator of Pgp3 and various chromosomal genes (11–13).

Three of the remaining six proteins are implicated in plasmid replication based on their amino acid sequences, while two are involved in plasmid maintenance. Specifically, Pgp1 is predicted to function as a DnaB helicase (14). Pgp5 and Pgp6 are expected to act as ParA and ParB, respectively, mediating the partitioning of daughter plasmids (15). Pgp7 and Pgp8, with homology to recombinase subunits XerC and XerD, are believed to mediate the resolution of plasmid multimers (15). Transformation studies have shown that plasmids lacking Pgp1, Pgp6, and Pgp8 fail to transform plasmid-free *C. trachomatis* and *C. muridarum*, whereas those lacking Pgp5 and Pgp7 do form transformants (12, 16). These findings indicate that Pgp1 and Pgp6 are essential for plasmid replication, Pgp8 is necessary for plasmid maintenance, and Pgp5 and Pgp7 are non-essential for replication and maintenance, likely due to functional redundancy with chromosome-encoded proteins.

In contrast to all the other plasmid-encoded proteins, the function of Pgp2 still needs to be discovered due to a lack of sequence homology with known proteins. In the aforementioned transformation studies, Pgp2-deficient plasmids also failed to transform *C. trachomatis* and *C. muridarum*, suggesting that Pgp2 is necessary for plasmid replication or maintenance (12, 16). In this study, we predicted the structure of Pgp2 using AlphaFold and identified it as a plasmid replication initiator through DALI protein three-dimensional (3-D) structural homology search. Additionally, we discovered a unique β-hairpin motif in Pgp2 that is absent in other replication initiators but highly conserved across *Chlamydia* species. An engineered plasmid lacking the β-hairpin motif (Pgp2Δ108–118) failed to transform *C. trachomatis*, providing strong evidence that Pgp2 functions as a plasmid replication initiator, with the conserved β-hairpin motif playing an essential role in its activity.

## RESULTS AND DISCUSSION

### Prediction of Pgp2 as a replication initiator

**Fig 1:**
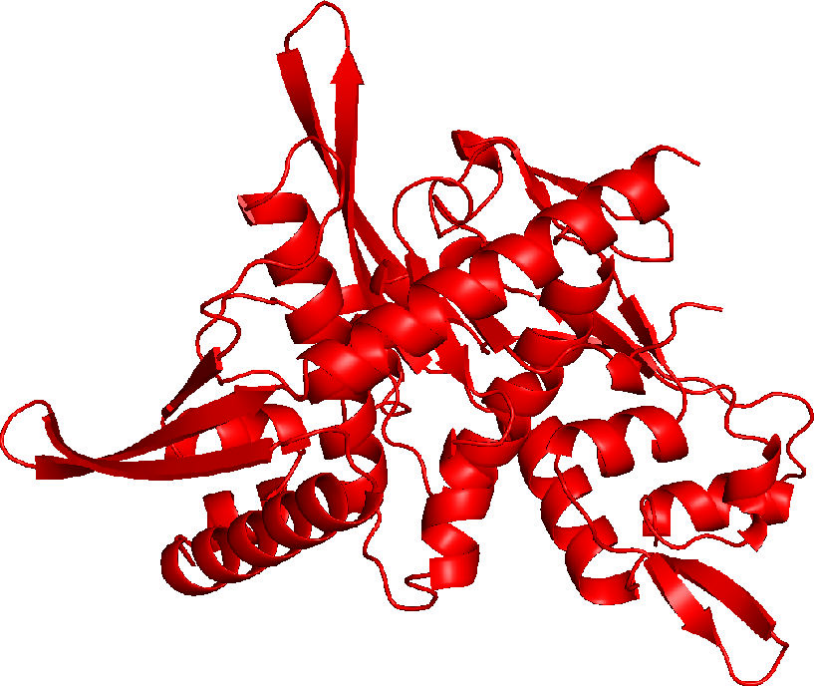
Three-dimensional structure of *C. trachomatis* L2 Pgp2. AlphaFold3 predicted the structure.

To determine the function of Pgp2, we first employed AlphaFold (17) to predict the 3-D structure of *C. trachomatis* Pgp2 (Fig. 1). The AlphaFold prediction yielded an average local distance difference test score of 90.24 out of a maximum score of 100, indicating a reliable structural model (17). We then identified structural homologs of Pgp2 using the DALI server (18), comparing the AlphaFold-predicted structure against a database of known protein structures. The DALI search revealed significant structural resemblances between the Pgp2 structure and experimentally determined structures of several known plasmid replication initiators, including the π initiator of the *Escherichia coli* R6K plasmid (PDB accession number 2NRA) (19), RepE of the *E. coli* F plasmid (PDB accession number 8AAN) (20), and RctB of *Vibrio cholerae* chromosome II (PDB accession number 5TBF), with root mean square deviation values of 3.3 Å, 3.3 Å, and 4.6 Å, respectively (Table 1). Notably, the *C. trachomatis* Pgp2 had Z-scores above 10 for all three matches, a DALI metric indicating how much the alignment score of a query structure with a target structure deviates from the mean score expected by chance. While a Z-score greater than 2.0 is generally considered significant for structural similarity, Z-scores above 8–10 represent highly similar and biologically relevant matches. These high Z-scores support the hypothesis that Pgp2 functions as a plasmid replication initiator, binding to its cognate DNA-binding site (iteron) sequences within the replication origin and recruiting the DNA helicase needed to initiate plasmid replication. The organization of Pgp1 (helicase) and Pgp2 as an operon further supports this functional prediction for Pgp2.

**TABLE 1:** Proteins with structural homology to the AlphaFold model of *C. trachomatis* Pgp2 identified by DALI search^*a*^

Rank	PDB accession # (reference)	Protein	Bacterium	Z-score	RMSD (Å)	lali	nres	Sequence identity (%)
1	2NRA (19)	π Initiator	*E. coli*	13.1	3.3	213	252	10
2	8AAN (20)	RepE	*E. coli*	12.7	3.3	205	228	8
3	5TBF	RctB	*V. cholerae*	10.8	4.6	209	280	10

^*a*^ The Z-score is an optimized similarity score defined as the sum of equivalent residue-wise Cα-Cα distances among two proteins. Abbreviations: RMSD, root mean square deviation of atomic positions; lali, length of alignment in two proteins; nres, the number of residues in the matched structure.

### Identification of a conserved β-hairpin motif in Pgp2 of *Chlamydia* species

**Fig 2:**
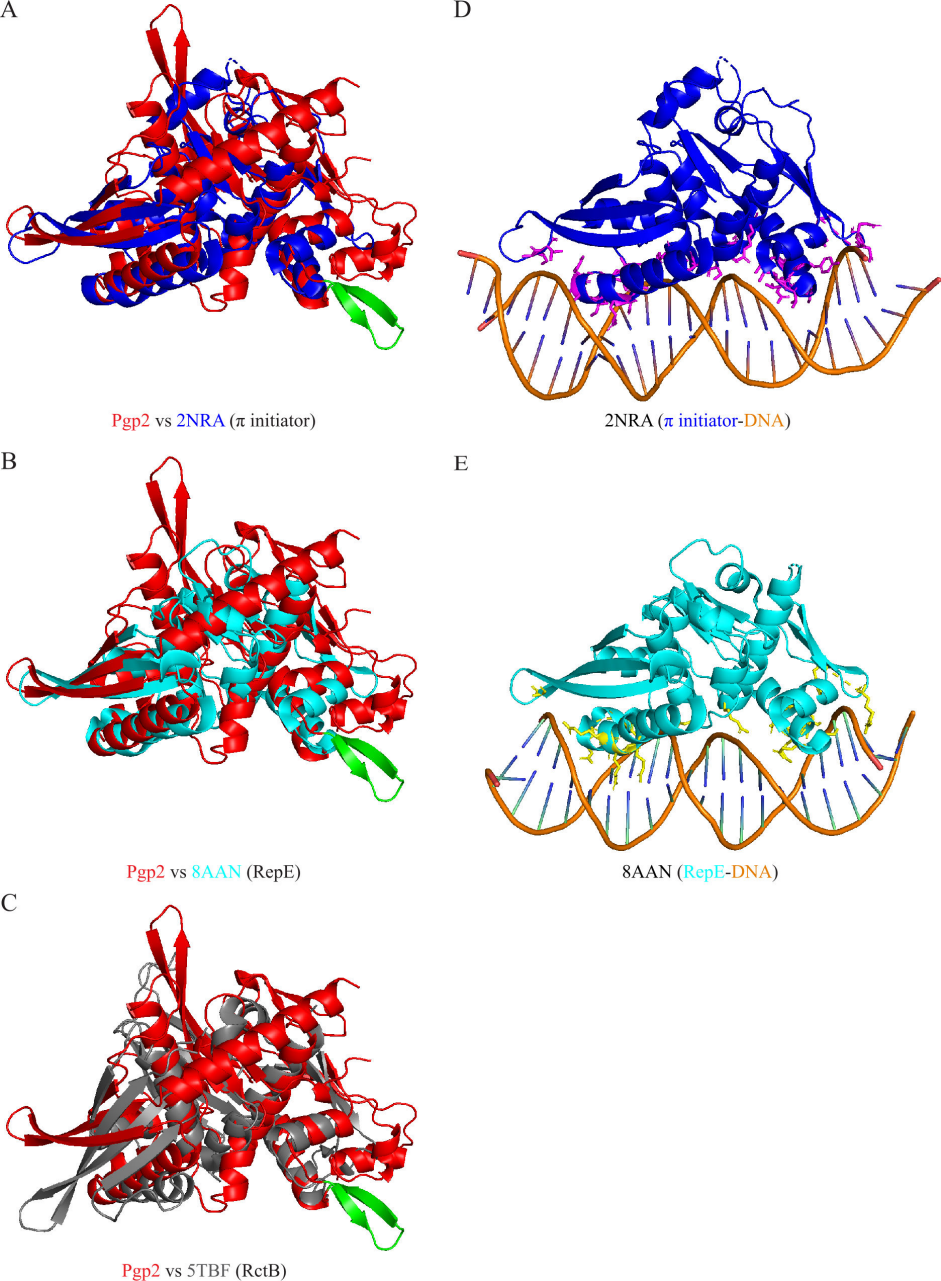
Identification of a β-hairpin motif extended from a helix whose equivalent in other replication initiators interacting with the iteron DNA. (**A–C**) Superimposition of AlphaFold-predicted Pgp2 structure (red) on the experimental structures of π initiator of the *E. coli* R6K plasmid (PDB accession number 2NRA) (blue), the RepE initiator of *E. coli* F plasmid (PDB accession number 8AAN (teal), and the RctB initiator of *V. cholerae* (PDB accession number 5TBF) (gray). Pgp2 features an 11-residue β-hairpin (green) not seen in other replication initiators. (**D, E**) Experimental structure of 2NRA and 8AAN containing respective iteron DNA. Note that a comparison of panels D and E with A and B, respectively, reveals that the unique β-hairpin in Pgp2 is an extension of a DNA-binding helix.

**Fig 3:**
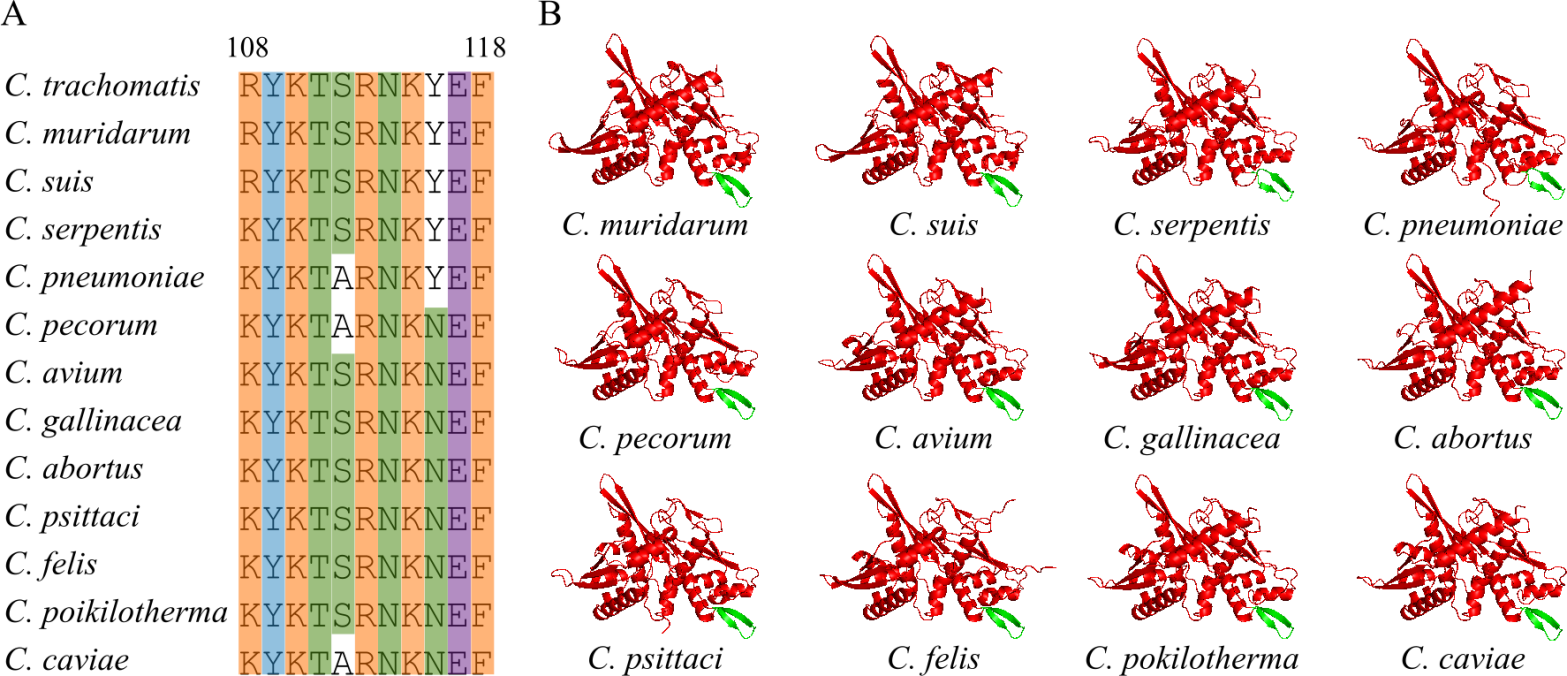
Conservation of the β-hairpin motif in Pgp2s of all *Chlamydia* species. (**A**) The amino acids constituting the β-hairpin in *C. trachomatis* Pgp2 are conserved in all 12 other species. Alignment of Pgp2s of all 13 *Chlamydia* species was performed using Clustal Omega, although only the 11 amino acid sequences that form the β-hairpin are presented. (**B**) Pgp2s of all 12 other *Chlamydia* species share the β-hairpin structure (green) with *C. trachomatis*. All structures were predicted using AlphaFold3.

Superimposition of the AlphaFold structure of *C. trachomatis* Pgp2 onto 2NRA, 8AAN, and 5TBF revealed a potentially distinct β-hairpin motif found only in Pgp2. This motif, highlighted in green in Fig. 2A through C, is absent in the other initiators. The β-hairpin motif in Pgp2 extends from a helix whose equivalents in 2NRA and 8AAN bind their respective iterons (Fig. 2D and E). The 11 residues comprising this motif form two short β-strands (R108-Y109-K110 and R113-N114-K115-Y116-E117-F118) connected by a two-residue loop (T111-S112) (Fig. 3A). Sequence alignment revealed that the residues constituting the β-hairpin motif in *C. trachomatis* Pgp2 are highly conserved across all 13 *Chlamydia* species (Fig. 3A); the same β-hairpin motif is present in the AlphaFold structures of all Pgp2s (Fig. 3B).

### Requirement of the β-hairpin motif for Pgp2 activity

**Fig 4:**
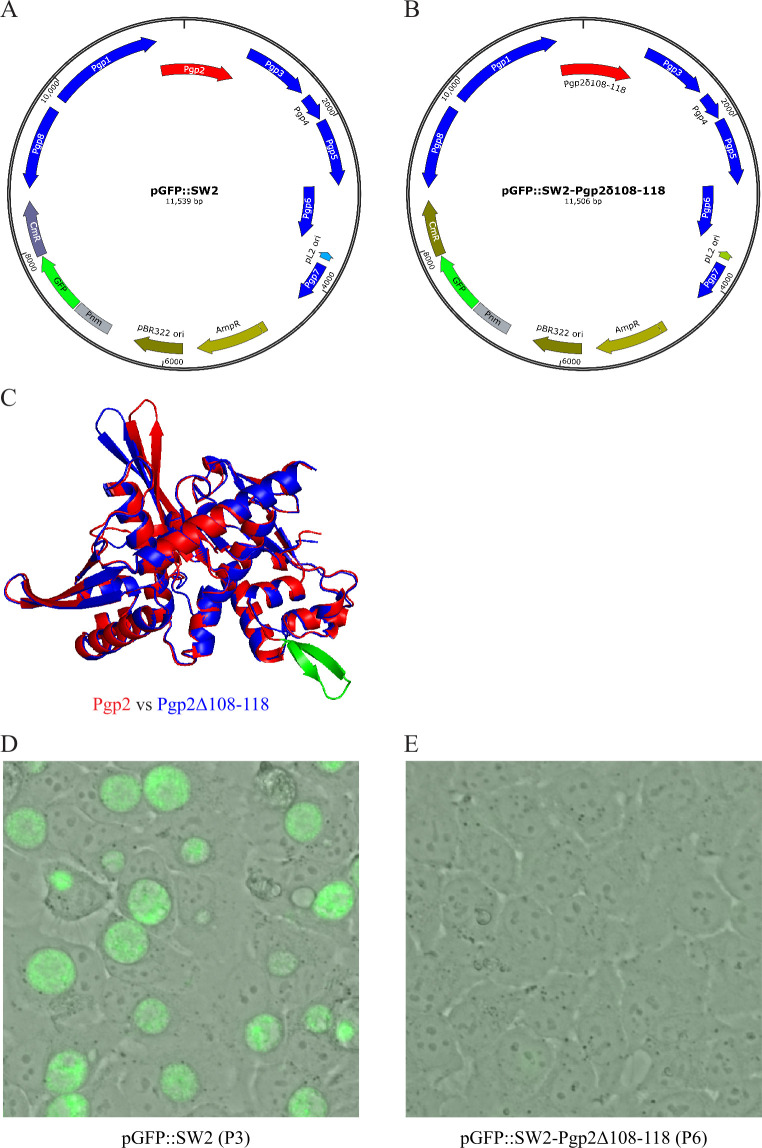
The distinct β-hairpin motif is required for Pgp2 activity. (**A**) The map of the shuttle vector pGFP::SW2 encoding wild-type Pgp2 (21). (**B**) The map of the shuttle vector pGFP::SW2- Pgp2Δ108–118 encoding the Pgp2 lacking residues 108–118 that form the unique β-hairpin. (**C**) Superimposition of wild-type Pgp2 and Pgp2Δ108–118. (**D**) A representative microscopic image of green fluorescence protein-expressing chlamydial inclusions formed in host cells in a passage 2 culture of *C. trachomatis* transformed with pGFP::SW2 and selected with ampicillin. (**E**) A representative microscopic image lacking fluorescence protein-expressing inclusions in a passage 6 culture of *C. trachomatis* transformed with pGFP::SW2-Pgp2Δ108–118 and selected with ampicillin. (**D, E**) Images were acquired at 36 hpi.

Given the conservation of the distinct β-hairpin motif identified through structural alignment near the predicted DNA-binding region, we hypothesized that the β-hairpin motif is required for Pgp2’s activity. To test this hypothesis, we removed the 33 nucleotides coding for R108-F118 of Pgp2 from pGFP::SW2, a shuttle vector containing the entire sequence of the *C. trachomatis* L2 plasmid and a green fluorescence protein (GFP) gene for easy detection of transformants (Fig. 4A and B) (21). Other than the absence of the β-hairpin motif, the AlphaFold structure of the modified Pgp2 (Pgp2Δ108–118) was fully superimposable with the wild-type Pgp2 structure, indicating that removing the β-hairpin did not affect the overall structure (Fig. 4C).

We transformed the plasmid-free *C. trachomatis* L2r strain (22) with pGFP::SW2 and pGFP::SW2-Pgp2Δ108–118. Transformants were selected by passaging cultures in a medium containing ampicillin as the selection agent. In all three independent transformation experiments, pGFP::SW2 transformants with green fluorescence were detectable starting at passage 2. In contrast, no pGFP::SW2-Pgp2Δ108–118 transformants were detected even by passage 5. Representative microscopic images of the pGFP::SW2 transformation culture with GFP-expressing chlamydiae at passage 3 and the pGFP::SW2-Pgp2Δ108–118 transformation culture lacking GFP signals at passage 6 are shown in Fig. 4D and E, respectively. These results suggest that the conserved β-hairpin motif is necessary for Pgp2’s function. Most likely, the β-hairpin motif contributes to DNA binding. Although the helix-turn-helix motif is more commonly associated with DNA binding, β-hairpin motifs have also been shown to bind DNA in some cases (e.g., references [23, 24]). Notably, 4 of the 11 amino acids in the β-hairpin are positively arginine and lysine residues (Fig. 3A); they likely facilitate interactions with the negatively charged iteron DNA, contributing to Pgp2’s binding specificity and function.

In conclusion, this study reveals that Pgp2, the sole function-unknown protein encoded by the virulence chlamydial plasmid, is a DNA replication initiator. We have demonstrated the requirement of a conserved β-hairpin motif for Pgp2’s function, highlighting the possibility of targeting this structure to disrupt plasmid-mediated virulence.

## MATERIALS AND METHODS

### Three-dimensional structure prediction

The amino acid sequences of Pgp2 from *Chlamydia* species, retrieved from the National Center for Biotechnology Information database, were used as inputs for structural prediction using the online AlphaFold3 servers (17). The top-ranked predicted structures were selected for further analysis.

### 3-D structure similarity analysis

The top-ranked AlphaFold-predicted structure of Pgp2 was submitted to the DALI server, and the resulting top hits were selected for superimposition. The superimposition was performed using PyMOL. The structural alignments were visually inspected to identify common structural motifs and unique features.

### Construction of vector expressing Pgp2 lacking the β-hairpin

pGFP::SW2-gp2Δ108–118 was constructed by assembling two overlapping DNA fragments amplified using the shuttle vector GFP::SW2 carrying all the eight Pgp genes (21) as the template. Fragment 1 was amplified using the forwarding primer named pgp2_cut_F1-F with the sequence 5´-gcttatggagttaagAGTGGAAAAGAAGCTGAAACT-3′ (the lowercase letters represent nucleotides coding for amino acids A102-K107 and the uppercase letters represent nucleotides coding for S119-T125) and the reverse primer US-pUC ori-F with the sequence 5´-gggattttggtcatgagattatc-3´ located in a noncoding sequence between the β-lactamase open reading frame and the pBR322 replication origin. Fragment 2 was amplified using the forwarding primer US-pUC ori-R with the 5´- GATAATCTCATGACCAAAATCCC-3′, which is reverse complementary to US-pUC ori-F, and reverse primer pgp2_cut_F2-R with the sequence 5′-CTTTTCCactcttaactccataagcctctaaga-3´ (the lowercase letters represent nucleotides that is reverse and complementary to codons 100–117 and uppercase letters represent nucleotides that are reverse complementary to codons 118 and 119 and part of codon 120). The Q5 high-fidelity DNA polymerase (New England Biolabs) was used to amplify the overlapping fragments, which were assembled using the NEBuilder HiFi DNA Assembly kit (New England Biolabs). NEB 10-beta competent *E. coli* was transformed with the assembly product. Transformed colonies were subject to PCR screening using diagnostic primers pgp2_cut_diag-F (5-ggagttaagAGTGGAAAA-3′) and pgp2_cut_diag-R (5´- taatcacccagtcgataaat-3´). Plasmid DNA from two colonies with positive amplification of the 391 bp diagnostic fragment was prepared and was subject to Nanopore whole plasmid sequencing at Quintara Biosciences (Boston). The plasmid with the confirmed deletion of codons 108–118 and without any additional mutations elsewhere was used to transform *Chlamydia*.

### *C. trachomatis* transformation

Transformation of the plasmid-free *C. trachomatis* variant L2r (22) with the parental shuttle vector pGFP::SW2 or its derivative pGFP::SW2-Pgp2Δ108–118 was performed as described (25) with modifications (26). Briefly, 10^7^ inclusion-forming units of elementary bodies were mixed with 5 µg of plasmid DNA in 50 µL CaCl_2_ buffer (10 mM Tris, pH 7.4, and 50 mM CaCl_2_) and incubated for 30 min at room temperature. The mixture was then diluted with Dulbecco’s modified Eagle’s medium containing 5% fetal bovine serum and 1 µg/mL cycloheximide and used to inoculate a six-well plate of confluent L929 cells (i.e., ~2 mL of the suspension per well). Monolayers were centrifuged at 900 × *g* for 30 min. Following incubation at 37 ℃ for 2 hours, ampicillin (sodium salt) was added to the cultures to a final concentration of 10 µg/mL. The passage 0 cultures were harvested at 36 hours post-infection (hpi) and passaged onto a new six-well plate at a 1:1 passage ratio using the centrifugation-assisted procedures described above, except that ampicillin was added to the cultures at the time of inoculation. Additional passages were performed every other day (25). Prior to passaging, cultures were examined under an Olympus IX51 fluorescence microscope for GFP-positive inclusions. Since pGFP::SW2 transformants are typically observed in passage 1 to passage 3 cultures, transformations with pGFP::SW2-Pgp2Δ108–118 were considered unsuccessful if GFP-expressing inclusions were not detected in passage 5 cultures.

### Imaging of *C. trachomatis* in host cells

HeLa229 cells grown on six-well plates were inoculated with harvests of a P2 culture of the pGFP::SW2 transformation or of a P5 culture of the pGFP::SW2Δ108–118. At 36 hpi, the culture medium was replaced with phosphate-buffered saline. Images were acquired on an Infinity i8-3 CMOS monochrome camera. Image pseudo-coloring and overlay were performed using the ACINST03 software (27).
